# The Occurrence of Myxomycete Communities in *Taxodium distichum* Forests: Influence of Substrates and Seasons

**DOI:** 10.3390/microorganisms12112156

**Published:** 2024-10-26

**Authors:** Yajing Chen, Wenlong Song, Gu Rao, Qun Dai, Shuzhen Yan, Shuanglin Chen

**Affiliations:** 1 School of Life Sciences, Nanjing Normal University, Nanjing 210023, China; chenyajing_0@163.com (Y.C.); wenlongsong2021@yeah.net (W.S.); raogufungi@163.com (G.R.); yanshuzhen@njnu.edu.cn (S.Y.); 2 School of Life Sciences and Chemical Engineering, Jiangsu Second Normal University, Nanjing 210013, China; njdaiqun@163.com

**Keywords:** plasmodial slime moulds, coniferous forest, species composition, species diversity, influencing factors

## Abstract

Myxomycete communities in *Taxodium distichum* forests and the influence of substrates and seasons on myxomycete diversity were studied. In total, 80 substrates (40 living barks and 40 litters) were collected from the sample site in winter, spring, summer and autumn of 2023, followed by moist chamber cultures. A total of 733 records representing 34 myxomycete species in 21 genera were obtained. *Arcyria cinerea* and six other species were abundant (A). Six species were common (C), six species were occasional (O), and fifteen species were rare (R). Species diversity (*H*’ = 2.04 ± 0.20) of corticolous communities was significantly higher than that (*H*’ = 1.49 ± 0.31) of litter-inhabiting communities (*p* < 0.05). Nonmetric Multidimensional Scaling (NMDS) analyses showed significant differences between these two communities (*p* < 0.01). Species diversity was slightly higher in summer and autumn compared to winter and spring. NMDS analyses indicated no significant differences among seasonal communities. Redundancy Analysis showed that the corticolous species were closely related to wind speed (*p* < 0.05), while litter-inhabiting species were mainly determined by temperature (*p* < 0.05). The study revealed that substrates and seasons influenced myxomycete communities in *T. distichum* forests, with the communities being particularly affected by the substrates in which they live.

## 1. Introduction

Myxomycetes (plasmodial slime moulds) are common eukaryotic microorganisms that generally occur in terrestrial ecosystems [1,2]. In the trophic stage, myxomycetes feed on bacteria, fungi and organic matter, acting as scavengers and contributing to nutrient cycling within ecosystems [3]. Although myxomycetes are widely distributed in terrestrial ecosystems, the most likely places to find them are moist temperate forests [4]. Thus, numerous studies on myxomycete diversity focus on forest ecosystems, with common substrates including rotten wood, bark, litter, herbivore feces and inflorescences [3,5]. Living bark and litter are typically the growth substrates for myxomycetes [6,7,8,9].

The occurrence of myxomycetes is closely related to vegetation [1,10]. There are different species compositions of the myxomycete community in various types of forests, and also there are differences in the species diversity and dominant species [11,12,13]. A mono forest is more favorable to study the changes in species composition and diversity in the myxomycete community because there are fewer influencing factors than a mixed forest. Previously, the species compositions of myxomycetes in several mono coniferous forests have been studied [14,15,16,17,18]. However, the knowledge provided with these studies was minimal. For example, Takahashi and Fukasawa [15] found only 17 myxomycete species from a *Cryptomeria japonica* forest in Japan.

There are many factors affecting myxomycetes communities and species diversity. Some studies have shown that they are affected by the substrates and their physicochemical properties including pH, water retention, electrical conductivity, and so on [15,19,20]. Climate factors such as temperature, precipitation, wind speed, and so on also affect the myxomycete communities by affecting the adhesion of spores, and the growth of plasmodia and fruiting bodies [5,21,22]. Changes in climate factors are accompanied by the alternation of the seasons, so the occurrence and community of myxomycetes change as seasons change. Tran et al. [23] pointed out that the peak abundance of field collection of myxomycetes shifted from the dry season to the rainy season in Thailand. Gao et al. [22] revealed that the richness and diversity of myxomycete species at the Baotianman National Nature Reserve of China were mainly influenced by sampling month. These all suggest that the myxomycete community is affected by climates in different seasons.

*Taxodium distichum* (L.) Rich. is a species of deciduous coniferous tree belonging to the family Taxodiaceae, native to North America. It is now found across several continents, including Asia, Africa, North America, South America, Europe and Oceania. This species of tree has also been introduced and planted in China, where it is widely planted in subtropical regions [24]. *T. distichum* thrives in warm, humid environments, particularly in wetlands and swamps. Hence, *T. distichum* forests can provide very favorable conditions for the occurrence of myxomycetes. Despite these favorable conditions, the community composition and species diversity of myxomycetes in *T. distichum* forests seem to be unknown. It is obviously necessary to conduct more comprehensive research on the myxomycete community, species diversity, and influencing factors in *T. distichum* forests. In this study, a sample site in a mono *T. distichum* forest was set up in Wuxiang Mountain National Forest Park, Nanjing, which is located in the north subtropical region of China. The substrates (living bark and litter) of *T. distichum* were collected in four seasons. Subsequently, moist chamber cultures were conducted to obtain myxomycete fruiting bodies. On the basis of the identification of myxomycetes species and the statistics of fruiting bodies, the species composition of myxomycete community was probed, the effects of substrates and seasons on the myxomycete community were analyzed, and substrate properties and climate factors affecting the species composition of the myxomycete community were explored.

## 2. Materials and Methods

### 2.1. Study Area and Substrate Collection

Wuxiang Mountain National Forest Park (118°59′33″–119°05′07″ E, 31°33′32″–31°36′58″ N) is situated in the suburb of Nanjing City, Jiangsu Province, China, with a total area of 62 square kilometers and an average elevation of about 100 m. Here, there is a North subtropical humid monsoon climate with four distinct seasons. The average annual temperature is 15.5 °C and the average annual precipitation is 1005.7 mm [25]. The study area is far away from industry, agriculture and residential areas, with less human interference, which may be more conducive to the occurrence of myxomycetes.

**Figure 1:**
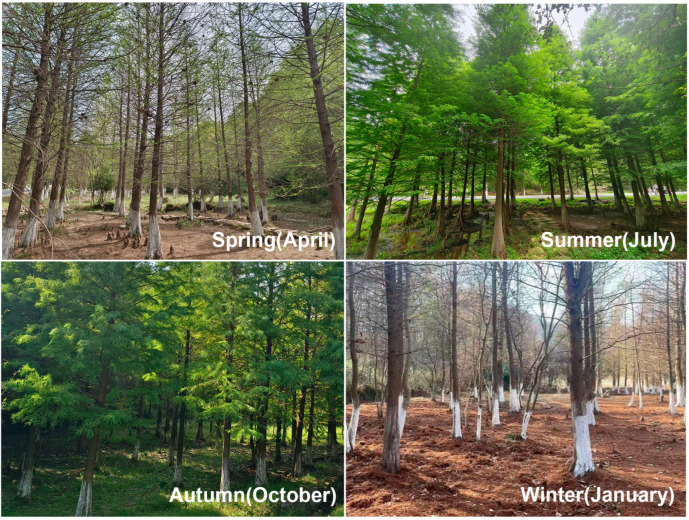
The sample site in Wuxiang Mountain National Forest Park, Jiangsu Province, China, across four seasons.

An 80 m × 20 sample site (119°01′23″ E, 31°35′42″ N) was set up in the forest of Wuxiang Mountain National Forest Park (Figure 1). These *T. distichum* trees are approximately 40 years old. Substrate samples were collected in winter (January), spring (April), summer (July) and autumn (October) of 2023. For each season, 10 sampling points were randomly selected in a “Z” pattern within the site to collect living barks at a height of approximately 1.5 m, and ground litter such as dead branches and fallen leaves under trees. Thus, a total of 80 substrate samples were obtained (20 samples per season: 10 from living barks and 10 from litters). The substrate samples were placed in separate sterile plastic bags and brought back to the laboratory for moist chamber cultures.

### 2.2. Moist Chamber Cultures

Moist chamber cultures were prepared following the method described by Li et al. [12]. Briefly, the substrate was placed in the Petri dish (9 cm × 9 cm) lined with sterile absorbent paper, with their outer surfaces facing up. Sterile distilled water at pH 7.0 was added to the dish until the substrate was submerged. After soaking for 24 h, the excess water was decanted. The dish was covered and placed in an HCS-250B constant temperature incubator (Tianjin Teste Instrument Co., Tianjin, China) for cultivation at 25 °C. All moist chambers were cultured for 60 d. During the cultivation process, sterile distilled water was regularly added to maintain the moisture of the substrate. Four moist chamber cultures were prepared for each substrate sample, resulting in a total of 320 cultures.

The occurrence of myxomycetes in the moist chambers was monitored under the JSZ6 dissecting microscope (Jiangnan Yongxin Optics Co., Nanjing, China) every day, and the mature fruiting bodies together with the substrate were taken from the moist chamber and put into the specimen box for dry preservation. The fruiting bodies of myxomycetes developed from the same plasmodium were counted as one record.

### 2.3. Identification of Myxomycete Species

All myxomycetes obtained were identified based on morphological characteristics mainly using Martin and Alexopoulos’ monograph [26]. Lado and Eliasson’s taxonomic system [27] was adopted. Lado’s nomenclature [28] was followed.

### 2.4. Determination of Some Influencing Factors

The influencing factors were divided into two categories: substrate properties (pH, electrical conductivity, water retention) and climate conditions (temperature, precipitation, wind speed). The determination of water retention of the substrate refers to the method used by Snell and Keller [29]. The substrates were dried, then weighed and recorded as M1. Subsequently, the substrates were placed in a Petri dish and immersed in 60 mL sterile distilled water for 24 h. After soaking, the substrates were removed, weighed, and recorded as M2. The calculation was WR = (M2 − M1)/M1, where WR refers to water retention. For the soaked liquid, the PHS-3E pH meter (INESA Scientific Instrument Co., Shanghai, China) was used to measure the pH value, and the DDS-11A electrical conductivity meter (INESA Scientific Instrument Co., Shanghai, China) was used to measure the electrical conductivity value. The climate condition parameters (temperature, precipitation, wind speed) of Wuxiang Mountain National Forest Park came from Jiangsu weather https://www.njgxjh.com (accessed on 24 March 2024). Table 1 summarizes these data.

### 2.5. Data Analysis

All data for this study were derived from myxomycete fruiting bodies obtained in moist chamber cultures. Moist chambers with fruiting bodies were designated as positive. The positive culture rate was calculated as the ratio of the number of moist chambers with fruiting bodies to the number of all moist chambers.

Based on the results of the identification of myxomycete species, the recorded numbers of each myxomycete species in two kinds of substrates (living bark and litter) and four seasons were counted. The ratio of the recorded numbers of a myxomycete species to the total recorded numbers was defined as relative abundance. The species abundance scale was classified following the criterion given by Stephenson et al. [30]: A: abundant (>3%); C: common (1.5–3%); O: occasional (0.5–1.5%); R: rare (<0.5%).

To assess the comprehensiveness of the investigation, we computed the Chao1 estimator [31] and plotted a species accumulation curve using EstimateS 9.1.0 software. The completeness of the investigation was determined by the ratio of the observed species count to the Chao1 estimator. The calculation was Chao1 = Sobs + n_1_(n_1_ − 1)/2(n_2_ + 1), where Sobs is the number of observed species, n_1_ is the number of species recorded only once, and n_2_ is the number of species recorded exactly twice.

The mean number of species per genus (S/G) was used to evaluate the taxonomic diversity [30]. The diversity characteristics of myxomycete communities were assessed using Shannon–Wiener diversity (*H*’), Species Evenness (*J*’), and Simpson diversity (*D*) [32]. These indices were computed using the “Vegan” R-package [33], and the one-way analysis of variance (ANOVA) and the independent samples *t*-tests were run using the GraphPad Prism 9.5.0 software.

The similarity of myxomycete communities across different substrates and seasons was measured using the Coefficient of Community (CC) and the Percentage Similarity (PS) [7]. The calculation of CC index was CC = 2c/(a + b), where a and b are the number of species in each assemblage, and c is the number of species common to both assemblages. The calculation of PS index was PS = ∑Min(a, b, c, …, x), where Min is the lower value of the relative abundance of species a, b, ..., x in the two communities. To further compare differences in myxomycete communities across two kinds of substrates and four seasons, the Bray–Curtis distances between samples were computed using the “Vegan” R-package [33]. Nonmetric Multidimensional Scaling (NMDS) ordination was applied to visualize sample clustering based on the Bray–Curtis distances.

To analyze the relationship between myxomycete communities and influencing factors, Redundancy Analysis (RDA) was conducted using the “Vegan” R-package [33]. Species coordinates near the center indicate strong adaptability to all influencing factors, coordinates at the edges suggest adaptation to specific environmental conditions, and similar coordinates imply similar growing conditions

## 3. Results

### 3.1. Species Composition of Myxomycete Communities

Myxomycete fruiting bodies were found and recorded in 187 moist chambers and 58.44% of the total 320 moist chambers. A total of 733 myxomycete records belonging to 34 species of 21 genera were obtained. Among them, 629 records belonging to 28 species of 18 genera occurred on living bark, 104 records belonging to 14 species of 13 genera occurred on litter, 186 records belonging to 13 species of 12 genera occurred in spring, 247 records belonging to 22 species of 16 genera occurred in summer, 169 records belonging to 20 species of 16 genera occurred in autumn and 131 records belonging to 20 species of 15 genera occurred in winter (Table 2). The Chao1 estimators generated for the complete dataset, the living bark, the litter, the spring, the summer, the autumn and the winter datasets were 40, 32, 17, 13, 27, 31 and 29, respectively. Thus, the estimated survey completeness for these seven assemblages was calculated as 85%, 88%, 82%, 100%, 81%, 65% and 67%, respectively (Table 2).

According to the abundance index (Table 3), seven species (*Clastoderma debaryanum*, *Physarum album*, *Cribraria confusa*, *Arcyria cinerea*, *Perichaena corticalis*, *Echinostelium minutum* and *Gulielmina vermicularis*) were abundant species (A), with relative abundances of more than 3%. Six species (*Trichia papillata*, *Ophiotheca chrysosperma*, *Paradiacheopsis longips*, *Comatricha nigra*, *Diderma chondrioderma* and *Hemitrichia minor*) were common species (C), with relative abundances of 1.5–3%. Six species (*Calomyxa metallica*, *Cribraria violacea*, *Ophiotheca pedata*, *Comatricha pulchelloides*, *Licea operculata* and *Stemonitis fusca*) were occasional species (O), with relative abundances of 0.5–1.5%. Fifteen species, such as *Comatricha pulchella*, *Comatricha elegans* and *Ceratiomyxa fruticulosa*, were rare species (R), with relative abundances of less than 0.5%.

### 3.2. Myxomycete Communities in Two Kinds of Substrates

**Figure 2:**
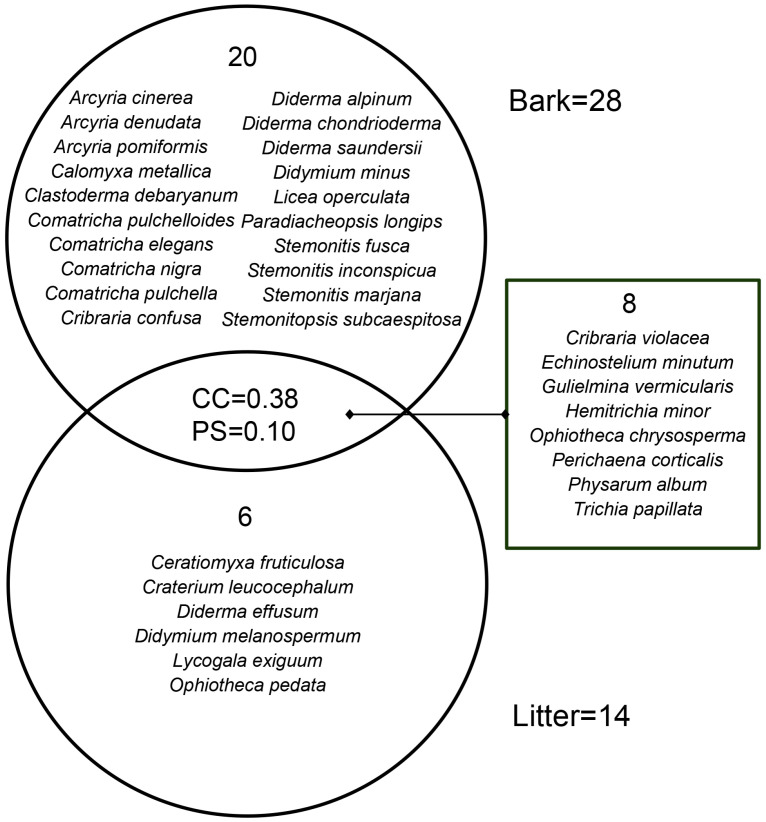
Venn diagram of the Coefficient of Community (CC) index, the Percentage Similarity (PS) index, and the species composition of myxomycete community on living bark and litter.

The positive culture rate of living bark moist chambers (88.80%) was much higher than that of litter moist chambers (28.13%). The corticolous myxomycete community had 28 species of 18 genera, and S/G was 1.56 (Table 2). In total, 20 species, such as *A. cinerea*, *C. metallica* and *C. debaryanum*, only occurred on living bark (Figure 2). The litter-inhabiting myxomycete community had 14 species of 13 genera, and S/G was 1.08 (Table 2). Six species (*C. fruticulosa*, *C. leucocephalum*, *D. effusum*, *D. melanospermum*, *L. exiguum* and *O. pedata*) occurred only on litter (Figure 2). Eight species (*C. violacea*, *E. minutum*, *G. vermicularis*, *H. minor*, *O. chrysosperma*, *P. corticalis*, *P. album* and *T. papillata*) occurred in two kinds of substrates (Figure 2). S/G of the litter-inhabiting myxomycete community was lower than that of the corticolous myxomycete community. It means that the litter-inhabiting myxomycete community has a higher taxonomic diversity because the biota in which the species are divided among many genera is intuitively more ‘diverse’ than one in which most species belong to only a few genera [34].

Among the corticolous myxomycete community, six species (*C. debaryanum*, *P. album*, *C. confusa*, *A. cinerea*, *E. minutum* and *P. corticalis*) were abundant species (A), four species (*P. longips*, *C. nigra*, *D. chondrioderma* and *C. metallica*) were common species (C), six species (*O. chrysosperma, H. minor*, *C. pulchelloides*, *L. operculata, S. fusca* and *C. pulchella*) were occasional species (O), and twelve species including *C. elegans*, *D. saundersii*, *T. papillata* and so on were rare species (R) (Table 3). Among the litter-inhabiting myxomycetes community, seven species (*G. vermicularis*, *P. corticalis*, *T. papillata*, *O. chrysosperma*, *C. violacea*, *O. pedata* and *H. minor*) were abundant species (A), two species (*C. fruticulosa* and *E. minutum*) were common species (C) and five species (*C. leucocephalum, D. effusum*, *D. melanospermum*, *L. exiguum* and *P. album*) were occasional species (O) (Table 3).

### 3.3. Myxomycete Communities in Four Seasons

**Figure 3:**
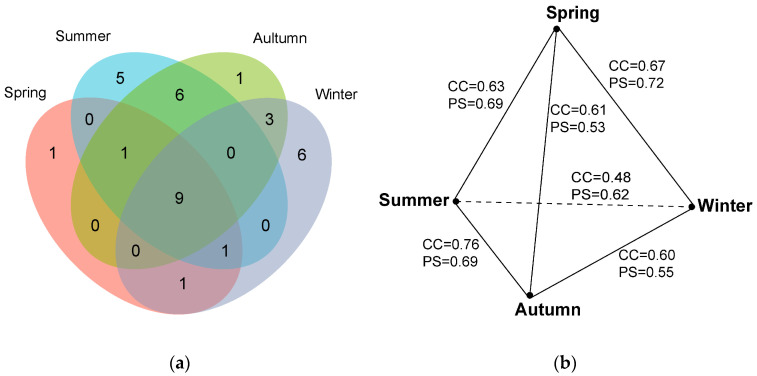
(**a**) Venn diagram of the species composition of myxomycetes in four seasons. (**b**) The relationship of CC and PS indices of the species composition of myxomycetes in four seasons.

The positive culture rate also varied across four seasons, following autumn (73.75%) > summer (65.00%) > winter (58.77%) > spring (46.25%). The myxomycete community in spring was composed of 13 species of 12 genera, and S/G was 1.08 (Table 2). *A. pomiformis* only occurred in spring (Table 3, Figure 3a). The myxomycete community in summer was composed of 22 species of 16 genera, and S/G was 1.38 (Table 2). Five species (*D. effusum*, *S. subcaespitosa*, *C. pulchella*, *C. leucocephalum* and *D. alpinum*) only occurred in summer (Table 3, Figure 3a). The myxomycete community in autumn was composed of 20 species of 16 genera, and S/G was 1.25 (Table 2). *C. fruticulosa* only occurred in autumn (Table 3, Figure 3a). The myxomycete community in winter was composed of 20 species of 15 genera, and S/G was 1.33 (Table 2). Six species (*A. denudata*, *D. melanospermum*, *S. inconspicua*, *D. minus*, *S. marjana* and *L. exiguum)* only occurred in winter (Table 3, Figure 3a). Nine species (*P. corticalis*, *O. chrysosperma*, *P. longipes*, *A. cinerea*, *E. minutum*, *P. album*, *T. papillata*, *C. debaryanum* and *H. minor*) occurred in all seasons (Table 3, Figure 3a).

In the myxomycete community in spring, four species (*C. confusa*, *C. debaryanum*, *P. album* and *A. cinerea*) were abundant species (A), four species (*P. longips*, *H. minor*, *O. chrysosperma* and *E. minutum*) were common species (C) and five species (*P. corticalis*, *S. fusca*, *T. papillata*, *A. pomiformi* and *G. vermicularis*) were occasional species (O) (Table 3). In the myxomycete community in summer, nine species (*C. debaryanum*, *A. cinerea*, *P. album*, *C. confusa*, *P. corticalis*, *E. minutum*, *C. nigra*, *G. vermicularis* and *C. violacea*) were abundant species (A), five species (*O. chrysosperma*, *T. papillata*, *P. longips*, *H. minor* and *C. metallica*) were common species (C), three species (*C. pulchella*, *C. pulchelloides* and *S. subcaespitosa*) were occasional species (O), and five species (*C. elegans*, *C. leucocephalum*, *D. alpinum*, *D. effusum* and *C. saundersi*) were rare species (R) (Table 3). In the myxomycete community in autumn, ten species (*C. debaryanum*, *P. album*, *G. vermicularis*, *P. corticalis*, *E. minutum*, *A. cinerea*, *D. chondrioderma*, *T. papillata*, *O. chrysosperma* and *C. metallica*) were abundant species (A), two species (*O. pedata* and *P. longips*) were common species (C) and eight species (*C. fruticulosa*, *C. elegans*, *C. nigra*, *C. pulchelloides*, *C. violacea*, *D. saundersii*, *H. minor* and *L. operculata*) were occasional species (O) (Table 3). In the myxomycete community in winter, seven species (*C. confusa*, *P. album*, *C. debaryanum*, *P. corticalis*, *E. minutum*, *A. cinerea* and *T. papillata*) were abundant species (A), four species (*L. operculata*, *P. longips*, *S. fusca* and *S. inconspicua*) were common species (C) and nine species (*A. denudata*, *D. chondrioderma*, *D. melanospermum*, *D. minus*, *H. minor*, *L. exiguum*, *O. chrysosperma*, *O. pedata* and *S. marjana*) were occasional species (O) (Table 3).

### 3.4. α Diversity and β Diversity of Myxomycete Community in Different Substrates and Seasons

**Figure 4:**
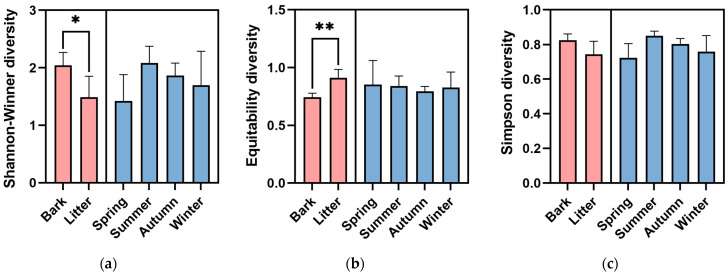
(**a**) Shannon–Wiener diversity, (**b**) Species Evenness, and (**c**) Simpson diversity of myxomycetes in two kinds of substrates and four seasons of the *Taxodium distichum* forest. The asterisk (*) denotes significance at the *p* < 0.05 level, and the two asterisks (**) denotes significance at the *p* < 0.01 level.

The Shannon–Wiener diversity of the corticolous myxomycetes (*H*’ = 2.04 ± 0.20) was significantly higher (*p* < 0.05) than that of the litter-inhabiting myxomycetes (*H*’ = 1.49 ± 0.31) (Figure 4a). Species Evenness of litter-inhabiting myxomycetes (*J*’ = 0.91 ± 0.06) was extremely significantly higher (*p* < 0.01) than that of corticolous myxomycetes (*J*’ *=* 0.74 ± 0.03) (Figure 4b), while the Simpson diversity did not vary significantly between the two kinds of substrates (Figure 4c).

**Figure 5:**
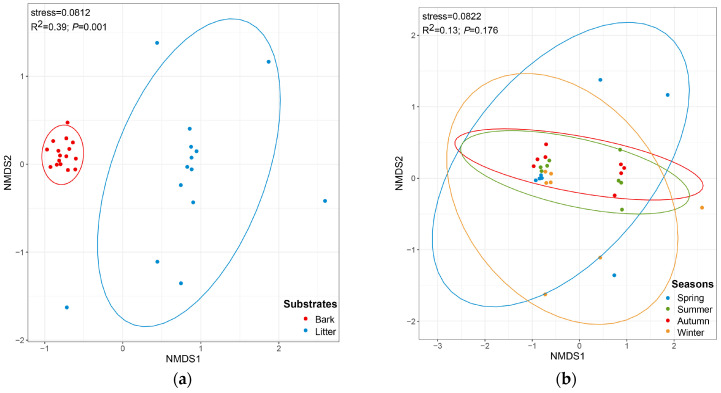
Non-metric multidimensional scaling plots, built on the Bray–Curtis distances of myxomycetes in two kinds of substrates (**a**) and four seasons (**b**).

The CC index and the PS index for the corticolous and litter-inhabiting myxomycete communities were 0.38 and 0.10, respectively (Figure 2). The NMDS ordination showed a good clustering effect (Figure 5a, stress = 0.0812) and revealed a significant difference between the corticolous and litter-inhabiting myxomycete communities (*p* = 0.001). The corticolous myxomycete communities exhibited closer relationships on the NMDS plot, whereas the litter-inhabiting myxomycete communities showed greater variation and divergence (Figure 5a). It may be related to the higher heterogeneity of litter than living bark.

The Shannon–Wiener diversity was the highest in summer (*H*’ = 2.08 ± 0.21), followed by autumn (*H*’ = 1.87 ± 0.15), winter (*H*’ = 1.70 ± 0.42), and spring (*H*’ = 1.42 ± 0.32) (Figure 4a). Species Evenness was the highest in spring (*J*’ = 0.85 ± 0.15), followed by summer (*J*’ = 0.84 ± 0.06), winter (*J*’ = 0.83 ± 0.09), and autumn (*J*’ = 0.79 ± 0.03) (Figure 4b). The Simpson diversity was the highest in summer (D = 0.85 ± 0.02), followed by autumn (D = 0.80 ± 0.02), winter (D = 0.76 ± 0.07), and spring (D = 0.72 ± 0.06) (Figure 4c). However, one-way ANOVA showed no significant differences in the Shannon–Wiener diversity, Species Evenness or Simpson diversity between four seasons (Figure 2).

The CC and PS indices were calculated to evaluate the similarities of the myxomycete communities between four seasons (Figure 3b). The highest CC index was 0.76 between the myxomycete communities in autumn and summer, followed by spring and winter (CC = 0.67), spring and summer (CC = 0.63), spring and autumn (CC = 0.61), autumn and winter (CC = 0.60), and winter and summer (CC = 0.48). The highest PS index was 0.72 between the myxomycete communities in spring and winter, followed by spring and summer (PS = 0.69), summer and autumn (PS = 0.69), summer and winter (PS = 0.62), winter and autumn (PS = 0.55), and spring and autumn (PS = 0.53) (Figure 3b).

The NMDS ordination indicated that the clustering effect of the myxomycete community was good (Figure 5b, stress = 0.0822), but there was no significant difference between the four seasons (*p* = 0.176). However, the NMDS ordination revealed that the myxomycete communities of summer and autumn were both relatively concentrated and showed a higher similarity. The myxomycete communities of winter and spring in *T. distichum* forests were more dispersed and also exhibited a higher similarity (Figure 5b). It may be related to the fact that summer and autumn are more suitable for the growth of myxomycetes than winter and spring.

### 3.5. Relationship between Myxomycete Communities and Influencing Factors

Based on the results above, it is clear that significant differences in the community composition and diversity exist between corticolous and litter-inhabiting myxomycetes in the *T. distichum* forest. Therefore, when examining the influence of environmental factors on myxomycete communities, it was essential to separate these two communities and evaluate the effects of some influencing factors on the composition of corticolous and litter-inhabiting myxomycetes separately.

**Figure 6:**
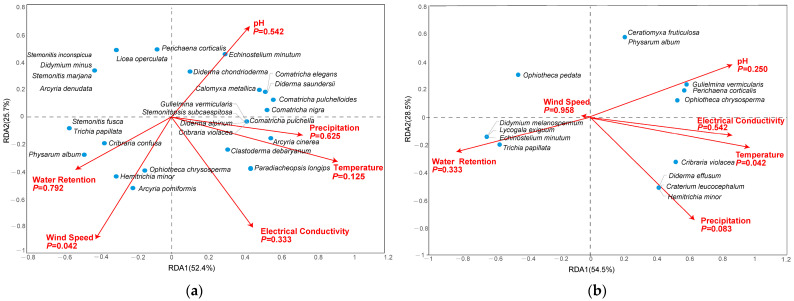
Redundancy Analysis plots of myxomycetes in relation to environmental factors in (**a**) corticolous myxomycetes and (**b**) litter-inhabiting myxomycetes.

The RDA indicated that wind speed had a significant influence (*p* < 0.05) on the corticolous myxomycete community, while pH, electrical conductivity, water retention, temperature, and precipitation had no significant influences (Figure 6a). For the litter-inhabiting myxomycete community, only temperature showed a significant influence (*p* < 0.05), while other factors, including pH, electrical conductivity, water retention, precipitation and wind speed, had no significant influences (Figure 6b).

The myxomycetes found in this study are mostly cosmopolitan and common species, which are less sensitive to environmental factors and have low requirements for environmental conditions. As a result, they do not cluster around any specific environmental factor in the RDA diagram but instead show a relatively scattered distribution.

In the corticolous myxomycetes, *C. metallica*, *C. elegans*, *D. chondrioderma*, *D. saundersii*, *E. minutum* and *P. corticalis* were significantly positively correlated with pH. Additionally, *A. cinerea*, *C. debaryanum*, *C. nigra*, *C. pulchella*, *C. pulchelloides*, *C. violacea*, *D. alpinum*, *G. vermicularis*, *P. longips*, and *S. subcaespitosa* were clearly positively correlated with precipitation and temperature. Regarding electrical conductivity, *A. cinerea*, *C. debaryanum* and *P. longips* showed clear positive correlations. For wind speed, *A. pomiformis*, *C. confusa*, *H. minor*, *O. chrysosperma* and *P. album* exhibited significant positive correlation. Lastly, *A. pomiformis*, *C. confusa*, *H. minor*, *O. chrysosperma*, *P. album*, *S. fusca* and *T. papillata* were obviously positively correlated with water retention (Figure 6a).

In the litter-inhabiting myxomycetes, *G. vermicularis*, *O. chrysosperma* and *P. corticalis* were significantly positively correlated with pH. Additionally, *C. leucocephalum*, *C. violacea*, *D. effusum*, *G. vermicularis*, *H. minor*, *O. chrysosperma* and *P. corticalis* were clearly positively correlated with both electrical conductivity and temperature. *C. leucocephalum*, *C. violacea*, *D. effusum* and *H. minor* showed clear positive correlations with precipitation. Lastly, *D. melanospermum*, *E. minutum*, *L. exiguum* and *T. papillata* were obviously positively correlated with precipitation and wind speed (Figure 6b).

## 4. Discussion

Moist chamber cultures have long been recognized as an effective method for obtaining myxomycete specimens and have been widely used in myxomycete studies [35]. In our study, the positive culture rate was 58.44%, which was similar to the results of some previous studies [12,36], indicating that the moist chamber cultures we used had a good effect. However, some studies have achieved higher positive culture rates, reaching over 95% [15,17]. In this study, the positive culture rate of living bark-moist chambers was 88.80%, which is a similar value to those from other studies in mono coniferous forests [15,16,17,18]. The positive culture rate for litter moist chambers was 28.13%, significantly lower than that of living bark. This result is consistent with that of Stephenson et al. [18]. This discrepancy in the positive culture rates between living bark and litter may be attributed to the characteristics of the coniferous litter, which is slender with a small surface area to receive myxomycete spores, and has a weak ability to retain spores. Conversely, rough coniferous bark is more conducive to adhering spores [37] and may retain more myxomycete spores than the litter.

In addition to the moist chamber, we found and collected seven species of larger myxomycetes in the field: *C. fruticulosa*, *A. cinerea*, *S. fusca*, *S. inconspicua*, *Stemonitis pallida*, *Stemonitis splendens* and *Collaria arcyrionema*. Notably, *S. pallida*, *S. splendens* and *C. arcyrionema* were not observed in moist chamber cultures. These seven myxomycete species were collected directly from the field only in summer, and no myxomycetes were found from the field in the other three seasons. The number of species collected from the field was also relatively low in the *T. distichum* forests. This may be due to the accumulation of high water levels on the floor of the *T. distichum* forest after rainfall. Field collections were typically conducted on litter and decayed wood on the forest floor, and the prolonged high water levels may inhibit the development of myxomycetes, thereby affecting the formation of fruiting bodies.

In coniferous trees, 34 species of myxomycetes were obtained from the *T. distichum* forests in this study, which was more than 17 species in the *C. japonica* forest [15], 23 species were associated with *Callitris* spp. [18], less than 44 species were obtained on the living bark of *M. glyptostroboides* [16] and 34 corticolous species were associated with 4 species of *Pinus* in different regions of the world [17]. This showed that myxomycetes in *T. distichum* forests may be more abundant and exhibit higher species diversity. Abundant species were *Comatricha rigida* and *Enerthenema berkeleyanum* from the *C. japonica* forest [15], *A. cinerea, C. confusa* and *D. effusum* from *Callitris* spp. [18], *D. chondrioderma*, *Licea variabilis* and *G. vermicularis* from *M. glyptostroboides* [16] and *E. minutum* and *Licea kleistobolus* from *Pinus* [17]. In the *T. distichum* forest, the most abundant species were *C. debaryanum* and *P. album.* This means that dominant species vary significantly in different coniferous forests. Additionally, the *T. distichum* forest only shared two species (*D. effusum* and *O. pedata*) with the *C. japonica* forest [15]. These all revealed that the species composition of the myxomycete community in the *T. distichum* forest was significantly different from those in other coniferous trees. Consequently, tree species may affect the composition of myxomycete communities.

In the *T. distichum* forest, 34 myxomycete species were obtained, with only 8 species shared between living bark and litter. The results of CC, PS and NMDS also indicate a significant difference between the corticolous and litter-inhabiting myxomycete communities (Figure 2 and Figure 5a). Additionally, abundant species (A) of the corticolous myxomycete community, such as *A. cinerea, C. debaryanum* and *C. confusa*, were not found on the litter. Abundant species (A) of litter-inhabiting myxomycete community, such as *O. pedata*, were not found on the living bark (Table 3). These results indicated that the substrate may significantly influence myxomycete composition, once again confirming the patterns observed in previous studies [38,39]. The differences may be attributed to the varying physicochemical properties and microbial communities, such as bacteria, on different substrates, which create distinct microhabitats. Different myxomycete species have varying requirements for growth and development, leading to substrate preferences [40].

NMDS results showed that the structure of corticolous myxomycete communities exhibited clear aggregation, whereas the litter-inhabiting myxomycete communities were more dispersed (Figure 5a). This may suggest that living bark, as a relatively stable substrate, has greater continuity and consistency in its properties across seasons, leading to smaller changes in the myxomycete communities. Litter, as a more heterogeneous substrate [40], may experience greater fluctuations in decomposition levels and organic matter content across seasons, resulting in more variation in the myxomycete communities and more dispersed distribution in the NMDS plot.

In the four seasons, the positive culture rates were higher in autumn and summer, exceeding 60%, while these were lower in spring and winter. The Shannon–Wiener index was found to be high in summer and autumn, and low in spring and winter (Figure 4a). Additionally, the PS and CC indices of myxomycete communities were higher between summer and autumn, as well as between spring and winter (Figure 3b). NMDS ordination also showed similar conclusions to those of the CC and PS indices and the myxomycete communities of summer and autumn were both relatively concentrated, while winter and spring were more dispersed (Figure 5b). This may be due to the low temperatures and low precipitation in spring and winter (Table 1). Myxomycete spores were less active in the field [5], and the myxomycete communities in the two seasons demonstrated a lower species diversity and positive culture rate. In summer, the high temperatures and abundant precipitation provided a stable and suitable environment for the growth of myxomycetes. Although precipitation decreases in autumn, the moderate temperatures still create suitable environmental conditions (Table 1). Spores matured and dispersed onto living bark and litter during the summer, allowing the same species to be collected in autumn. Therefore, the community structure in summer and autumn was similar and concentrated, and positive culture rates and species diversity were higher (Figure 3b and Figure 5b). Similarly, Takahashi and Hada [10] found that litter-inhabiting myxomycetes in temperate forests of Japan exhibited clear seasonal occurrence patterns. Fruiting bodies began to appear in April, with the most active sporulation period from June to September, peaking in July. In our study, this peak sporulation period also corresponds to the summer (July) characterized by warm and humid climate conditions. Ko Ko et al. [41] observed that the occurrence and species diversity of myxomycetes were influenced by the season, with higher species composition and diversity in warm and wet seasons compared to cold and dry seasons. Our results are also consistent with the above patterns: the occurrence and species diversity of myxomycetes exhibit distinct seasonal regularity, with warm and humid environments being more conducive to their growth and development.

The RDA results demonstrated the relationships between myxomycete species and environmental factors. Among the corticolous myxomycete community, six species were identified as abundant (A), each exhibiting distinct correlations with environmental factors that reflected their ecological preferences. *C. debaryanum* and *A. cinerea* were positively associated with temperature and precipitation, suggesting these species thrived in environments with higher warmth and moisture levels. *P. album* and *C. confusa* showed positive correlations with water retention and wind speed, indicating a preference for microhabitats that retained moisture and have sufficient air circulation, which helps prevent desiccation and supports spore dispersal. Additionally, *E. minutum* and *P. corticalis* were positively correlated with pH, implying that they favored less acidic environments (Figure 6a). Similarly, among the litter-inhabiting myxomycetes, seven abundant species were identified. *G. vermicularis*, *P. corticalis*, and *O. chrysosperma* were positively associated with pH and temperature, indicating a preference for warmer, less acidic environments. *C. violacea* and *H. minor* showed positive correlations with precipitation and temperature, suggesting they were more abundant in moist and warmer conditions. Finally, *T. papillata* and *O. pedata* were positively correlated with water retention, reflecting their adaptation to microhabitats with higher moisture levels (Figure 6b). These results were similar to those of previous studies. For example, *E. minutum* was positively correlated with pH, *A. cinerea* and *C. debaryanum* were positively correlated with temperature, and *C. violacea* was positively correlated with precipitation [12,22]. These findings underscored the ecological diversity among myxomycetes, revealing their distinct environmental preferences and potential sensitivity to environmental changes.

The RDA results showed that the temperature had a significant effect (*p* < 0.05) on the litter-inhabiting myxomycete community in the *T. distichum* forest (Figure 6b). Temperature, as an important climatic factor, has been highlighted in numerous previous studies on myxomycetes. For instance, Li et al. [12] indicated that temperature was an important climate factor affecting litter-inhabiting myxomycetes. Takahashi and Hada [10] demonstrated that the occurrence of litter-inhabiting myxomycete species was closely related to the changes in mean and minimum temperature. The results of our study are consistent with the rules shown in the above studies. Temperature is speculated to influence myxomycete nutrient supply by altering litter decomposition rates mediated by bacteria, fungi and other microorganisms [42,43]. In summary, both substrate properties (pH, electrical conductivity, water retention) and climate factors (temperature, precipitation, wind speed) influence the seasonal changes in myxomycete composition in the *T. distichum* forest. Among these factors, temperature and wind speed exert particularly pronounced effects.

## 5. Conclusions

This study examined the myxomycete communities in the *T. distichum* forest, focusing on the influence of substrates and seasons. In total, 28 species were found in corticolous communities and 14 species in litter-inhabiting communities. CC and PS values between the two communities were 0.38 and 0.10, respectively, and NMDS analyses revealed clear differences. RDA showed that wind speed significantly influenced corticolous communities and temperature played a key role in litter-inhabiting communities. Although species diversity was slightly higher in summer and autumn compared to winter and spring, NMDS analysis revealed no significant seasonal differences overall. These findings suggested that while seasonal variations may have exerted some influence, they were not the primary factor shaping the structure of myxomycete communities. Substrate types appeared to be more dominant in determining community composition and diversity.

## Figures and Tables

**Table 1:** Substrate properties and climate conditions at the sampling site of *Taxodium distichum* forest.

			Spring	Summer	Autumn	Winter
Substrate properties	Living bark	pH	5.09 ± 0.49	5.77 ± 0.45	5.43 ± 0.59	5.59 ± 0.44
		Electrical conductivity (μs/cm)	122.17 ± 10.11	107.36 ± 12.10	110.16 ± 19.97	79.23 ± 9.31
		Water retention (%)	224 ± 90	206 ± 67	123 ± 34	223 ± 48
	Litter	pH	7.09 ± 0.09	7.24 ± 0.12	7.34 ± 0.08	6.95 ± 0.19
		Electrical conductivity (μs/cm)	217.47 ± 24.55	219.47 ± 38.72	183.73 ± 29.10	128.2 ± 14.73
		Water retention (%)	231 ± 24	139 ± 50	117 ± 39	249 ± 36
Climate conditions		Temperature (°C)	15.83 ± 7.01	27.17 ± 4.14	18.33 ± 7.06	4.50 ± 4.92
		Precipitation (mm)	126.2	669.6	81.7	67.6
		Wind speed (km/h)	12.84 ± 1.08	11.08 ± 1.32	10.91 ± 0.83	11.13 ± 0.82

**Table 2:** The number of records, species and genera, the Chao1 estimator, the completeness of the survey, and the mean number of species per genus (S/G) of myxomycetes in two kinds of substrates and four seasons of the *Taxodium distichum* forest.

	Substrates		Sampling Seasons				Overall
	Living Bark	Litter	Spring	Summer	Autumn	Winter	
The number of records	629	104	186	247	169	131	733
The number of species	28	14	13	22	20	20	34
The number of genera	18	13	12	16	16	15	21
Chao1	32	17	13	27	31	29	40
Completeness	88%	82%	100%	81%	65%	67%	85%
S/G	1.56	1.08	1.08	1.38	1.25	1.33	1.62

**Table 3:** Abundance indices of myxomycetes for two kinds of substrates and four seasons of the *Taxodium distichum* forest.

Species	Substrates		Sampling Seasons				Overall
	Living Bark	Litter	Spring	Summer	Autumn	Winter	
*Arcyria cinerea*	11.8%		9.7%	14.2%	8.3%	5.3%	A
*Arcyria denudata*	0.2%					0.8%	R
*Arcyria pomiformis*	0.2%		0.5%				R
*Calomyxa metallica*	1.6%			1.6%	3.6%		O
*Ceratiomyxa fruticulosa*		1.9%			1.2%		R
*Clastoderma debaryanum*	22.1%		25.3%	16.6%	21.9%	10.7%	A
*Comatricha elegans*	0.3%			0.4%	0.6%		R
*Comatricha nigra*	2.1%			4.9%	0.6%		C
*Comatricha pulchella*	0.5%			1.2%			R
*Comatricha pulchelloides*	0.6%			1.2%	0.6%		O
*Craterium leucocephalum*		1.0%		0.4%			R
*Cribraria confusa*	19.2%		28.0%	12.1%		29.8%	A
*Cribraria violacea*	0.2%	7.7%		3.2%	0.6%		O
*Diderma alpinum*	0.2%			0.4%			R
*Diderma chondrioderma*	2.1%				7.1%	0.8%	C
*Diderma effusum*		1.0%		0.4%			R
*Diderma saundersii*	0.3%			0.4%	0.6%		R
*Didymium melanospermum*		1.0%				0.8%	R
*Didymium minus*	0.2%					0.8%	R
*Echinostelium minutum*	6.7%	1.9%	1.6%	6.1%	9.5%	7.6%	A
*Gulielmina vermicularis*	0.2%	26.9%	0.5%	4.5%	10.1%		A
*Hemitrichia minor*	1.1%	3.8%	2.2%	2.0%	0.6%	0.8%	C
*Licea operculata*	0.6%				0.6%	2.3%	O
*Lycogala exiguum*		1.0%				0.8%	R
*Ophiotheca chrysosperma*	1.3%	9.6%	2.2%	2.8%	3.6%	0.8%	C
*Ophiotheca pedata*		4.8%			2.4%	0.8%	O
*Paradiacheopsis longips*	2.5%		2.7%	2.4%	1.8%	1.5%	C
*Perichaena corticalis*	4.6%	22.1%	1.1%	8.5%	9.5%	9.9%	A
*Physarum album*	19.9%	1.0%	24.2%	13.0%	13.6%	19.8%	A
*Stemonitis fusca*	0.6%		1.1%			1.5%	O
*Stemonitis inconspicua*	0.3%					1.5%	R
*Stemonitis marjana*	0.2%					0.8%	R
*Stemonitopsis subcaespitosa*	0.3%			0.8%			R
*Trichia papillata*	0.3%	16.3%	1.1%	2.8%	3.6%	3.1%	C

## Data Availability

The raw data supporting the conclusions of this article will be made available by the authors upon request.

## References

[1] Ndiritu G.G., Spiegel F.W., Stephenson S.L. (2009). Distribution and ecology of the assemblages of myxomycetes associated with major vegetation types in Big Bend National Park, USA. Fungal Ecol..

[2] Schnittler M., Dagamac N.H.A., Woyzichovski J., Novozhilov Y.K., Stephenson S.L., Rojas C.A. (2022). Biogeographical patterns in myxomycetes. Myxomycetes: Biology, Systematics, Biogeography, and Ecology.

[3] Novozhilov Y.K., Rollins A.W., Shchepin O.N., Schnittler M., Stephenson S.L., Rojas C.A. (2022). Ecology and distribution of Myxomycetes. Myxomycetes: Biology, Systematics, Biogeography, and Ecology.

[4] Alexopoulos C.J., Mims C.W., Blackwell M. (1996). Introductory Mycology.

[5] Stephenson S.L. (2023). Past and ongoing field-based studies of Myxomycetes. Microorganisms.

[6] Nguyen L.T.T., Sanchez-Mahecha O., Almadrones-Reyes K.J., Redena-Santos J.C., Dagamac N.H.A. (2019). Occurrence of leaf litter inhabiting myxomycetes from lowland forest patches of Northern and Central Vietnam. Trop. Ecol..

[7] Stephenson S.L. (1989). Distribution and ecology of myxomycetes in temperate forests II: Patterns of occurrence on bark surface of living trees, leaf litter, and dung. Mycologia.

[8] Vaz A.B.M., Santos D.S.D., Cardoso D., van den Berg C., de Queiroz L.P., Badotti F., Fonseca P.L.C., Cavalcanti L.H., Goes-Neto A. (2017). Corticolous myxomycetes assemblages in a seasonally dry tropical forest in Brazil. Mycoscience.

[9] Walker L.M., Cedeno-Sanchez M., Carbonero F., Herre E.A., Turner B.L., Wright S.J., Stephenson S.L. (2019). The response of litter associated Myxomycetes to long-term nutrient addition in a lowland tropical forest. J. Eukaryot. Microbiol..

[10] Takahashi K., Hada Y. (2012). Seasonal occurrence and distribution of myxomycetes on different types of leaf litter in a warm temperate forest of western Japan. Mycoscience.

[11] Trevino-Zevallos I.F., Lado C. (2020). Myxomycete diversity in a humid montane forest on the eastern slopes of the Peruvian Andes. Plant Ecol. Evol..

[12] Li M., Tao X., Li B., Du Q., Zhu X.Q., Huang D.M., Yan S.Z., Chen S.L. (2021). Spatiotemporal distribution and dynamic changes of myxomycetes in subtropical forests of China. Fungal Ecol..

[13] Stephenson S.L., Novozhilov Y.K., Shchepin O.N., Laursen G.A., Leontyev D.V., Schnittler M. (2022). Myxomycetes of Alaska: Species diversity and distribution. Nova Hedwig..

[14] Takahashi K., Harakon Y., Fukasawa Y. (2018). Geographical distribution of myxomycetes living on *Cryptomeria japonica* bark in Japan. Mycoscience.

[15] Takahashi K., Fukasawa Y. (2022). Association between corticolous myxomycetes and tree vitality in *Cryptomeria japonica*. Mycoscience.

[16] Takahashi K. (2024). Myxomycetes on the bark of living *Metasequoia glyptostroboides* trees and their distribution along a rural-urban gradient. Mycoscience.

[17] Stephenson S.L., Marbaniang T.M., Gupta P., Rojas C. (2020). Assemblages of corticolous myxomycetes associated with species of *Pinus* (Pinaceae) in four different regions of the world. Nova Hedwig..

[18] Stephenson S.L., Elliott T.F., Elliot K., Vernes K. (2023). Myxomycetes associated with the bark, cones and leaves of Australian cypress pines (*Callitris* spp.). Aust. J. Bot..

[19] Schnittler M., Unterseher M., Tesmer J. (2006). Species richness and ecological characterization of myxomycetes and myxomycete-like organisms in the canopy of a temperate deciduous forest. Mycologia.

[20] Everhart S.E., Keller H.W., Ely J.S. (2008). Influence of bark pH on the occurrence and distribution of tree canopy myxomycete species. Mycologia.

[21] Schnittler M., Erastova D.A., Shchepin O.N., Heinrich E., Novozhilov Y.K. (2015). Four years in the Caucasus–observations on the ecology of nivicolous myxomycetes. Fungal Ecol..

[22] Gao Y., Yan S.Z., Wang G.W., He G., Chen S.L. (2018). Myxomycete diversity and ecology in the Baotianman National Nature Reserve, a subtropical mountain forest in central China. Fungal Ecol..

[23] Tran H.T.M., Stephenson S.L., Hyde K.D., Mongkolporn O. (2006). Distribution and occurrence of myxomycetes in tropical forests of northern Thailand. Fungal Divers..

[24] Fu L.G., Yu Y.F., Mill R.R., Wu Z.Y. (1999). Taxodiaceae. Flora of China v.4 Cycadaceae through Fagaceae.

[25] Deng S.Q., Guan Q.W., Yan J.F., Yu S.Q., Ma C.H. (2009). Characterization of community structure and adjustment of stand structure in Wuxiang Mountain National Forest Park in Nanjing. J. Chin. Urban For..

[26] Martin G.W., Alexopoulos C.J. (1969). The Myxomycetes.

[27] Lado C., Eliasson U.H., Stephenson S.L., Rojas C.A. (2022). Taxonomy and systematics: Current knowledge and approaches on the taxonomic treatment of Myxomycetes. Myxomycetes: Biology, Systematics, Biogeography, and Ecology.

[28] Lado C. (2024). An Online Nomenclatural Information System of Eumycetozoa. Real Jardín Botánico, CSIC, Madrid, Spain. http://www.nomen.eumycetozoa.com.

[29] Snell K.L., Keller H.W. (2003). Vertical distribution and assemblages of corticolous myxomycetes on five tree species in the Great Smoky Mountains National Park. Mycologia.

[30] Stephenson S.L., Kalyanasundaram I., Lakhanpal T.N. (1993). A comparative biogeographical study of myxomycetes in the mid-Appalachians of eastern North America and two regions of India. J. Biogeogr..

[31] Chao A. (1984). Nonparametric estimation of the number of classes in a population. Scand. J. Stat..

[32] Kaper H.G., Roberts F.S. (2019). Mathematics of Planet Earth: Protecting Our Planet, Learning from the Past, Safeguarding for the Future.

[33] Oksanen J., Simpson G.L., Blanchet F.G., Kindt R., Legendre P., Minchin P.R., O’Hara R.B., Solymos P., Stevens M.H.H., Szoecs E. (2024). Vegan: Community Ecology Package, R package version 2.6–8; CRAN. https://CRAN.R-project.org/package=vegan.

[34] Simberloff D.S. (1970). Taxonomic diversity of island biotas. Evolution.

[35] Bordelon A.P., Keller H.W., Scarborough A.R. (2024). An inexpensive moist chamber cultures technique for finding microbiota on live tree bark. Appl. Plant Sci..

[36] Dagamac N.H.A., dela Cruz T.E.E., Pangilinan M.V.B., Stephenson S.L. (2011). List of species collected and interactive database of myxomycetes (plasmodial slime molds) for Mt. Arayat National Park, Pampanga Philippines. Mycosphere.

[37] Takahashi K. (2014). Influence of bark characteristics on the occurrence of corticolous myxomycetes in western Japan. J. Jpn. Bot..

[38] Macabago S., Stephenson S.L., dela Cruz T.E.E. (2016). Diversity and distribution of myxomycetes in coastal and mountain forests of Lubang Island, Occidental Mindoro, Philippines. Mycosphere.

[39] Li M., Gao Y., Yao L., Wang G.W., Yan S.Z., Chen S.L. (2022). Distribution characteristics of myxomycetes among substrates, study areas, and forest types in central China. Mycol. Prog..

[40] Stephenson S.L. (2011). From morphological to molecular: Studies of myxomycetes since the publication of the Martin and Alexopoulos (1969) monograph. Fungal Divers..

[41] Ko Ko T.W., Stephenson S.L., Hyde K.D., Lumyong S. (2011). Influence of seasonality on the occurrence of myxomycetes. Chiang Mai J. Sci..

[42] Anderson J.M. (1991). The effects of climate change on decomposition processes in grassland and coniferous forests. Ecol. Appl..

[43] Fierer N., Craine J.M., Schimel M.L.P. (2005). Litter quality and the temperature sensitivity of decomposition. Ecology.

